# Pyrolytic and Kinetic Analysis of Two Coastal Plant Species: *Artemisia annua* and *Chenopodium glaucum*

**DOI:** 10.1155/2013/162907

**Published:** 2013-11-19

**Authors:** Lili Li, Xiaoning Wang, Jinsheng Sun, Yichen Zhang, Song Qin

**Affiliations:** ^1^Yantai Institute of Coastal Zone Research, Chinese Academy of Sciences, 17 Chunhui Road, Laishan District, Yantai 264003, China; ^2^Tianjin Key Laboratory of Animal and Plant Resistance/College of Life Science, Tianjin Normal University, Tianjin 300387, China; ^3^Tianjin Aquatic Animal Infectious Disease Control and Prevention Center, Tianjin 300221, China

## Abstract

The large amount of coastal plant species available makes them ideal candidates for energy production. In this study, thermogravimetric analysis was used to evaluate the fuel properties of two coastal plant species, and the distributed activation energy model (DAEM) was employed in kinetic analysis. The major mass loss due to devolatilization started at 154 and 162°C at the heating rate of 10°C min^−1^ for *Artemisia annua* and *Chenopodium glaucum*, respectively. The results showed that the average activation energies of *Artemisia annua* and *Chenopodium glaucum* were 169.69 and 170.48 kJ mol^−1^, respectively. Furthermore, the activation energy changed while the conversion rate increased, and the frequency factor *k*_0_ decreased greatly while the activation energy decreased. The results also indicated that the devolatilization of the two coastal plant species underwent a set of first-order reactions and could be expressed by the DAEM. Additionally, a simplified mathematical model was proposed to facilitate the prediction of devolatilization curves.

## 1. Introduction

Biomass is one of the most promising feedstocks for biofuel production because of its merits of renewability and wide distribution. *Artemisia annua* and *Chenopodium glaucum* are coastal plant species that can adapt to several stressful environmental conditions, including high saline content, drought, and heavy metal pollution. They are annual halophytes and distributed widely in North America, Europe, Africa, and Asia. *Artemisia annua* and *Chenopodium glaucum* obtained our interests because of their high yields, appropriate biomass characteristics, low input demands, and positive environmental impacts, such as helping to improve the soil texture and reduce soil salinity. The large amount of these plant species available makes them ideal candidates for energy production.

Among various energy conversion technologies, pyrolysis is one of the most commonly used techniques, which is characterized by thermal conversion of biomass into useful fuel at high temperature in the absence of oxidizing atmosphere [1]. Furthermore, thermogravimetric analysis is a highly precise method for the study of pyrolysis, and it is shown that each kind of biomass has unique pyrolysis characteristics because of its specific proportions of the components [2]. Pyrolytic and kinetic data from thermogravimetry analysis not only are useful for understanding the processes and mechanisms of the thermal degradation but also can be used as input parameters for a thermal degradation reaction system [3].

Numerous models have been used for the pyrolytic analysis. However, it has been found that distributed activation energy model (DAEM) is more accurate than some pseudomechanistic models, especially when the decomposition is carried out under inert atmosphere [4–6]. The present study has evaluated the fuel properties of *Artemisia annua* and *Chenopodium glaucum*. The kinetic parameters were determined by the DAEM method, and a simplified mathematical model was proposed to facilitate the prediction of devolatilization curves.

## 2. Materials and Methods

### 2.1. Materials

*Artemisia annua* and *Chenopodium glaucum* were collected from a coastal zone of Yantai, Shandong province, China. The plants were oven-dried at 60°C to constant weight and then grounded with a Mini-Mill to pass through a 125 *μ*m sieve.

### 2.2. Proximate and Ultimate Analysis

The moisture analysis was conducted according to ASTM E871-82 (2006). The ash content was determined according to ASTM E1755-01 (2007). The volatile matter content was analyzed according to ASTM E872-82 (2006). The fixed carbon was expressed as the 100%-ash content-volatile matter-moisture content. The C, H, O, N, and S contents in the samples were measured according to our previous study [7]. All measurements were replicated three times.

### 2.3. Thermogravimetric Analysis

The powders of *Artemisia annua* and *Chenopodium glaucum* were analyzed by a Mettler Toledo TGA/DSC1 STARe thermoanalyzer. The pyrolysis experiments were performed at heating rates of 5, 10, 20, and 30°C min^−1^ in a dynamic high purity nitrogen flow of 50 mL min^−1^. The temperature of the furnace was programmed to rise from room temperature to 900°C.

### 2.4. Kinetic Analysis Using DAEM

DAEM has been widely used in analyzing the thermal reaction system of biomasses [6, 8]. It assumes that many irreversible first-order parallel reactions that have different rate parameters occur simultaneously. The model is expressed as

(1) $$\begin{array}{c} 1-\frac{m_{t}}{m_{0}}=\int_{0}^{\infty }\exp\left(-k_{0}\int_{0}^{t}\exp\left(-\frac{E}{RT}\right)\text{d}t\right)f\left(E\right)\text{d}E, \end{array}$$

where *m*_*t*_ is the mass loss by time *t*, *m*_0_ is the total mass loss at the end of pyrolysis, *f*(*E*) is the distribution function of activation energy *E* that represents the difference in the activation energies of many first-order parallel reactions, and *k*_0_ is the frequency factor corresponding to *E* value. After a series of reduction, including transformation and approximation [9], DAEM can be expressed as

(2) $$\begin{array}{c} \ln\left(\frac{\beta }{T^{2}}\right)=\ln\left(\frac{k_{0}R}{E}\right)+0.6075-\frac{E}{RT}. \end{array}$$

Equation (2) establishes a linear relationship between ln⁡(*β*/*T* ^2^) and (1/*T*) with the slope of (−*E*/*R*), where *β* is the heating rate and *R* is the universal gas constant. Activation energy *E* and frequency factor *k*_0_ can be determined by the slope and intercept of the Arrhenius plots.

## 3. Results and Discussion

### 3.1. Proximate and Ultimate Analysis

Proximate and ultimate analysis facilitates the correlation of the individual composition to the thermal behavior of the biomass materials. Proximate analysis showed that the ash contents of *Artemisia annua* and *Chenopodium glaucum* were 4.4 and 5.1 wt%, respectively. *Artemisia annua* was a little lower in ash content than *Chenopodium glaucum* (Table 1). Ultimate analysis showed that these coastal plant species consisted of moderately high contents of carbon and oxygen and low amounts of nitrogen, hydrogen, and sulfur (Table 1).

### 3.2. Characterization of Pyrolysis

**Figure 1:**
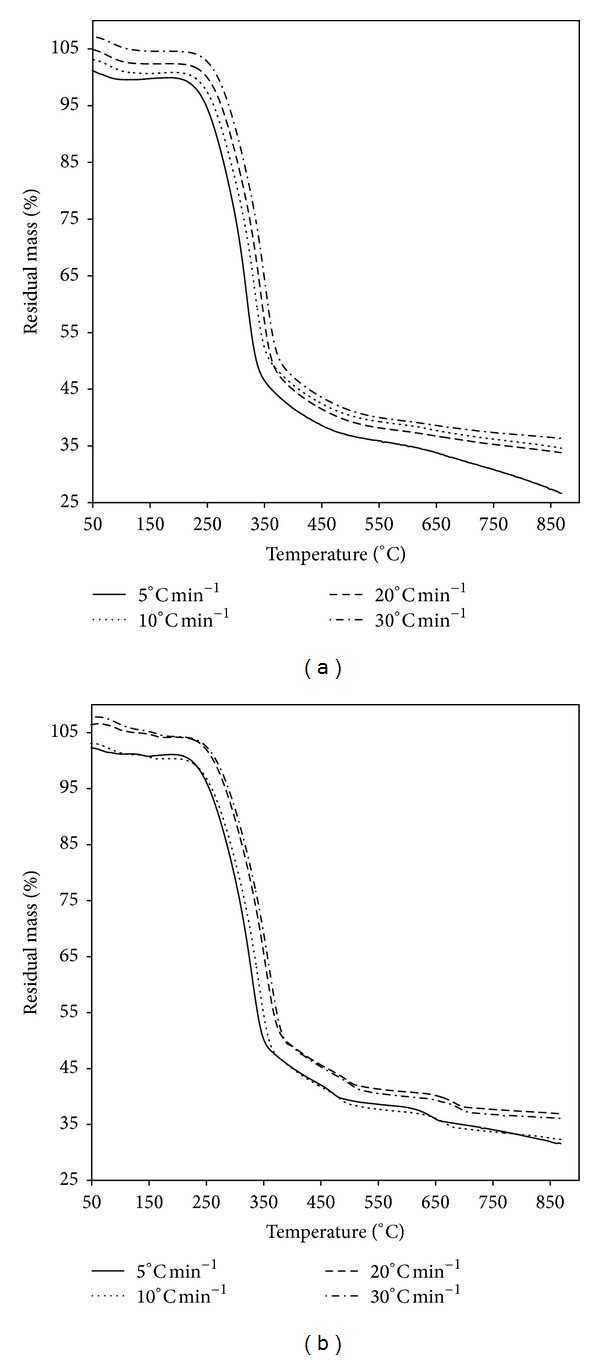
TG curves for *Artemisia annua* (a) and *Chenopodium glaucum* (b).

**Figure 2:**
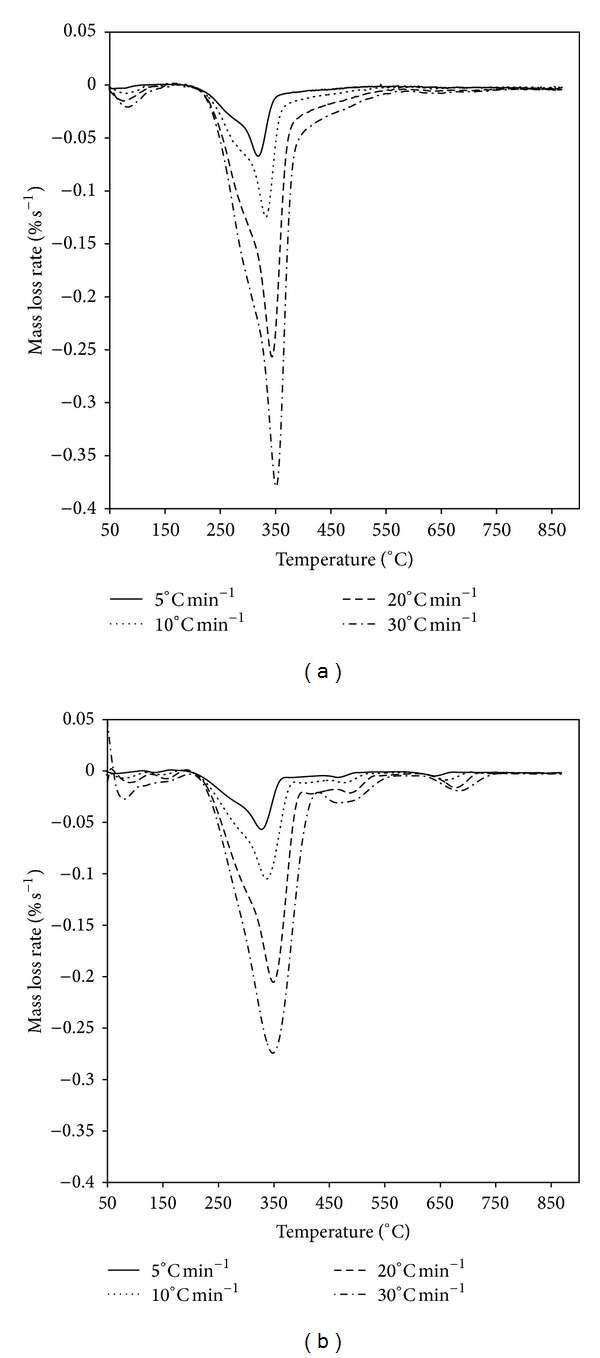
DTG curves for *Artemisia annua* (a) and *Chenopodium glaucum* (b).

The shape of the thermo-gravimetry (TG) curves did not change with different heating rates. However, TG curves shifted toward the right while the heating rate increased (Figure 1). This was due to the effect that the time to reach a given temperature became shorter by an increased heating rate and caused the entire curve to shift to higher temperatures. The temperatures corresponding to the maximum mass loss rate were also increased with the increasing of heating rate (Table 2). Additionally, the maximum rate of decomposition tended to increase at higher heating rate because there was more thermal energy to facilitate better heat transfer between the surroundings and the insides of the samples (Figure 2).

Table 2 showed characteristics of the thermal degradation for the two coastal plant species at different heating rates. The average rate of mass loss at the same heating rate for *Artemisia annua* was higher than that for *Chenopodium glaucum*, which indicated that *Artemisia annua* had a little higher pyrolysis reactivity. Furthermore, the major mass loss due to devolatilization started at lower temperature for *Artemisia annua* than *Chenopodium glaucum*. At the heating rate of 10°C min^−1^, for example, the onset of devolatilization occurred at 154 and 162°C for *Artemisia annua* and *Chenopodium glaucum*, respectively.

### 3.3. Kinetic Analysis

**Figure 3:**
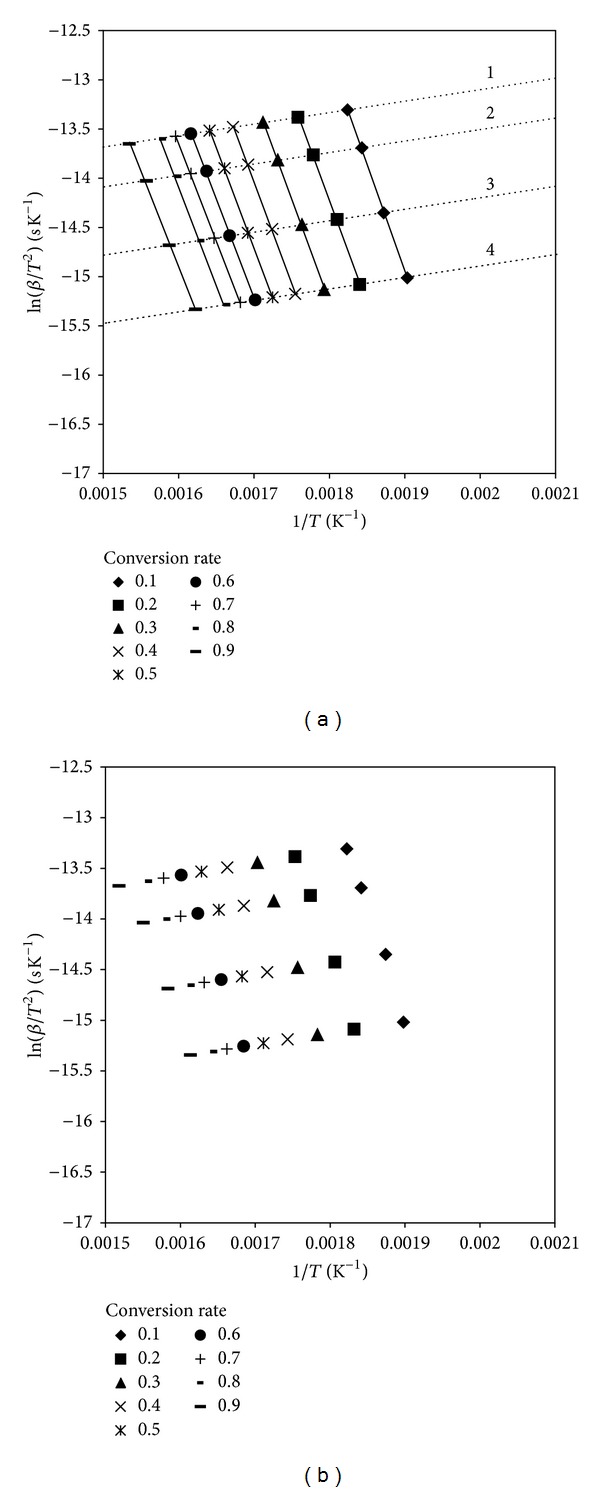
Determination of the temperature at which devolatilization occurred for *Artemisia annua* (a) and *Chenopodium glaucum* (b). The dotted lines of 1, 2, 3, and 4 were at the heating rates of 5, 10, 20, and 30°C min^−1^, respectively.

The linear and parallel development for different conversion rates from 0.1 to 0.9 at various heating rates was shown in Figure 3. All plots had fairly high linear correlation coefficients (*R* ^2^ ≥ 0.98), indicating that the devolatilization of two plant species underwent a set of first-order reactions. The results showed that *Artemisia annua* and *Chenopodium glaucum* were fairly similar in the average activation energies, which were 169.69 and 170.48 kJ mol^−1^, respectively (Table 3).

While the conversion rate increased from 0.1 to 0.9, the *E* value of *Artemisia annua* decreased from 180.03 to 161.45 kJ mol^−1^, whereas that of *Chenopodium glaucum* decreased from 185.84 to 147.17 kJ mol^−1^ (Table 3). It can be seen that the frequency factor *k*_0_ decreased greatly while *E* values decreased. For *Artemisia annua*, for example, the *k*_0_ value decreased from 2.82 × 10^12^ to 1.12 × 10^8^ s^−1^ when the *E* value decreased from 180.03 to 161.45 kJ mol^−1^.

### 3.4. Prediction of Devolatilization Curves

When Figure 3 was available, a simplified mathematical model could be established to predict the devolatilization curves. The full line in Figure 3(a) showed that the data points obtained for each particular devolatilization rate could be linearized. And the dotted line in Figure 3(a) showed that the data points obtained for each heating rate could also be linearized, which could be described in the form of *y* = *a*(1/*T*) + *b* + ln⁡*β* (where *a* was the slope of the line and *b* + ln⁡*β* was the intercept of the line) [10]. The simplified process was exhibited in Table 4. Then the temperature at which devolatilization occurred can be determined with the intersection between the linearization for a heating rate and for each devolatilization rate. *T* can be described in

(3) $$\begin{array}{c} T=\frac{E/R+1166.55}{\ln\left(k_{0}R/E\right)+15.35-\ln\left(\beta \right)}. \end{array}$$

**Figure 4:**
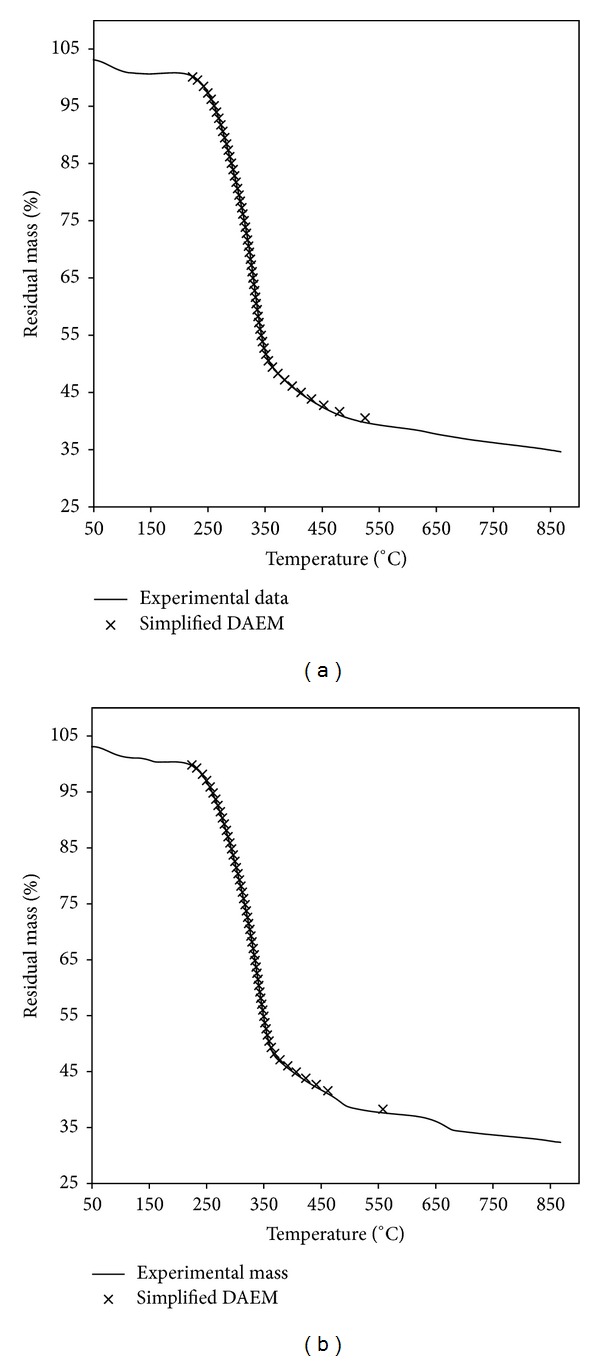
Prediction of the TG curves for *Artemisia annua* (a) and *Chenopodium glaucum* (b).

**Figure 5:**
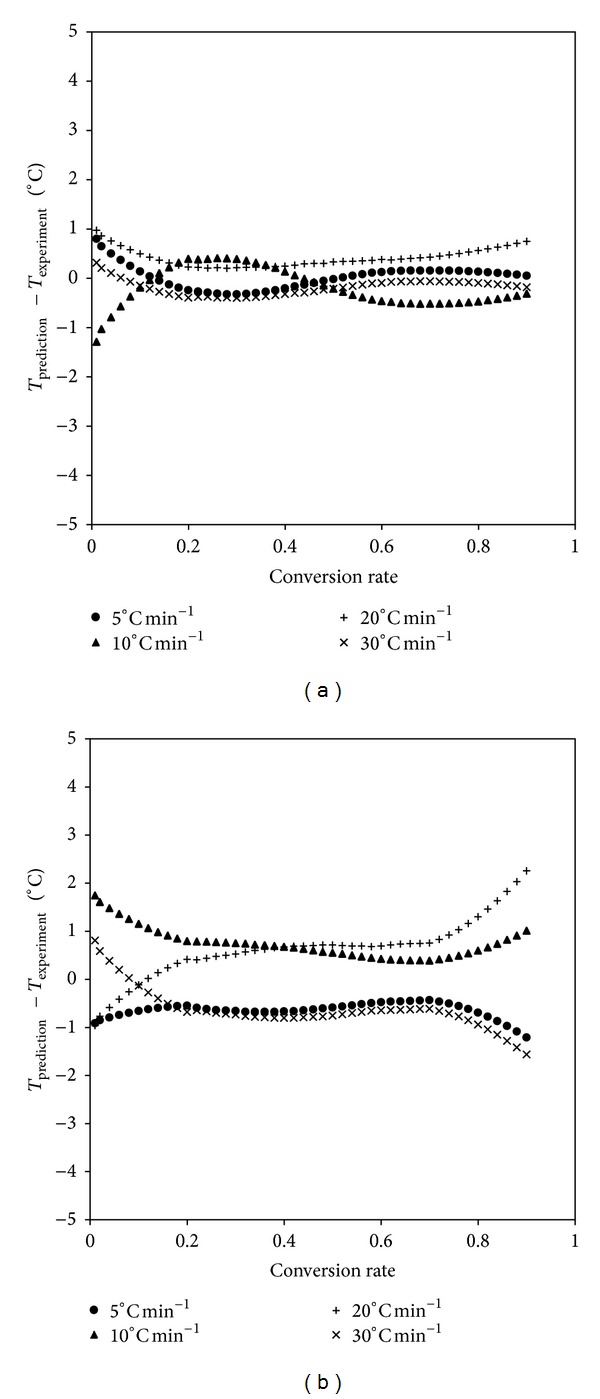
Difference between experimental temperature and predicted temperature for *Artemisia annua* (a) and *Chenopodium glaucum* (b).

The experimental devolatilization curves at the heating rate of 10°C min^−1^ were plotted together with the curves determined by the simplified mathematical model (Figure 4), which matched the experimental data very well for the two coastal plant species. The results were similar for the heating rate of 5, 20 and 30°C min^−1^. Moreover, the maximum relative errors between the experimental temperature and those obtained by the simplified mathematical model were 0.85% and 0.88% for *Artemisia annua* and *Chenopodium glaucum*, respectively (Figure 5). From this validation, it was evident that the developed simplified mathematical model closely predicted the devolatilization curves of *Artemisia annua* and *Chenopodium glaucum*. Therefore, the simplified mathematical model is a useful tool for the prediction of devolatilization curves.

## 4. Conclusions

The fuel properties of *Artemisia annua* and *Chenopodium glaucum* were evaluated. The major mass loss due to devolatilization started at 154 and 162°C at the heating rate of 10°C min^−1^ for *Artemisia annua* and *Chenopodium glaucum*, respectively. The results showed that average activation energies of *Artemisia annua* and *Chenopodium glaucum* were 169.69 and 170.48 kJ mol^−1^, respectively. The activation energy changed while the conversion rate increased, and the frequency factor *k*_0_ decreased greatly while activation energy decreased. The results also indicated that the devolatilization of the two coastal plant species underwent a set of first-order reactions and could be expressed by the DAEM. Additionally, a simplified mathematical model was proved to be a credible tool for the prediction of devolatilization curves.

## Figures and Tables

**Table 1:** Proximate and ultimate analysis of *Artemisia annua* and *Chenopodium glaucum*.

Analysis	Properties/%	*Artemisia annua *	*Chenopodium glaucum *
Proximate analysis	Water content	2.97 ± 0.036	3.10 ± 0.090
	Volatile	81.32 ± 3.520	79.95 ± 1.728
	Ash	4.41 ± 0.038	5.10 ± 0.115
	Fixed carbon	11.30	11.85

Ultimate analysis	C	44.45 ± 0.005	42.77 ± 0.021
	H	6.26 ± 0.001	6.03 ± 0.035
	N	0.76 ± 0.004	0.86 ± 0.036
	S	Not Detected	Not Detected
	O	31.31 ± 0.007	35.45 ± 0.014

**Table 2:** Characteristics of pyrolysis for *Artemisia annua* and *Chenopodium glaucum*.

Species	Heating rate/°C min^−1^	*T*_*i*_/°C^a^	*T*_*f*_/°C^b^	*T*_1_/°C^c^	(−d*α*/dt)_average_/% s^−1^	(−d*α*/dt)_max_/% s^−1^	Mass loss/%^d^
*Artemisia annua *	5	140	396	318	0.0070	0.0672	73.35
	10	154	410	333	0.0135	0.1247	65.41
	20	167	433	344	0.0252	0.2566	66.18
	30	173	448	351	0.0363	0.3794	63.65

*Chenopodium glaucum *	5	148	391	330	0.0065	0.0568	68.47
	10	162	401	337	0.0128	0.1049	67.67
	20	172	420	353	0.0238	0.2054	63.10
	30	179	465	360	0.0358	0.2742	63.91

^a^ *T*_*i*_ was the initial temperature of the major mass loss stage.

^b^ *T*_*f*_ was the final temperature of the major mass loss stage.

^c^ *T*_1_ was the temperature corresponding to the larger peak of the DTG curve.

^d^Mass loss = (Initial mass − Residue mass)/Initial mass × 100%.

**Table 3:** Kinetic parameters analyzed by DAEM for *Artemisia annua* and *Chenopodium glaucum*.

Convertion rate	*Artemisia annua *			*Chenopodium glaucum *		
	*E*/kJ mol^−1^	*k*_0_/s^−1^	*R* ^2^	*E*/kJ mol^−1^	*k*_0_/s^−1^	*R* ^2^
0.1	180.03	2.82 × 10^12^	1.00	185.84	1.03 × 10^13^	0.99
0.2	173.17	1.44 × 10^11^	1.00	178.55	4.20 × 10^11^	0.99
0.3	173.72	5.81 × 10^10^	1.00	176.15	8.19 × 10^10^	0.99
0.4	171.54	1.54 × 10^10^	1.00	175.49	2.91 × 10^10^	1.00
0.5	169.66	5.30 × 10^9^	1.00	171.24	5.73 × 10^9^	1.00
0.6	166.57	1.67 × 10^9^	1.00	169.97	2.44 × 10^9^	1.00
0.7	164.73	7.48 × 10^8^	1.00	167.81	9.65 × 10^8^	1.00
0.8	166.29	6.53 × 10^8^	1.00	162.11	1.94 × 10^8^	1.00
0.9	161.45	1.12 × 10^8^	1.00	147.17	5.71 × 10^6^	0.98

Average	169.69			170.48		

**Table 4:** Derivation of prediction equation.

Line	Equation: *y* = *a*(1/*T*) + *b* + ln*β*	*a*	*b*
1	*y* = 1193.22(1/*T*) − 14.78 + ln⁡⁡(30/60)	1193.22	−14.78
2	*y* = 1178.49(1/*T*) − 14.76 + ln⁡⁡(20/60)	1178.49	−14.76
3	*y* = 1158.18(1/*T*) − 14.73 + ln⁡(10/60)	1158.18	−14.73
4	*y* = 1136.31(1/*T*) − 14.69 + ln⁡(5/60)	1136.31	−14.69

Average		1166.55	−14.74
